# Point of choice kilocalorie labelling in the UK eating out of home sector: a descriptive study of major chains

**DOI:** 10.1186/s12889-019-7017-5

**Published:** 2019-05-28

**Authors:** Eric Robinson, Sam Burton, Tom Gough, Andrew Jones, Ashleigh Haynes

**Affiliations:** Institute of Psychology, Health & Society, University of Liverpool, Liverpool, L69 7ZA UK

**Keywords:** Kilocalorie labelling, Food environment, Eating out, Restaurant food, Nutrition information, Obesity, Kcals

## Abstract

**Background:**

Eating out is now common and food served out of the home is often of low nutritional quality. Kilocalorie (kcal) labelling of food and drink products sold in restaurant chains in the US is now mandatory, although in store kcal labelling practices among major UK restaurant and takeaway chains have not been examined.

**Methods:**

During August 2018, we contacted, visited the website and/or retail outlets of major eating out and takeaway food chains in the UK, including full-service and fast-food restaurants, cafes and coffee shops, some of which had previously made a voluntary pledge to provide kcal labelling. We examined the proportion of chains providing kcal information to customers at point of choice in store and the extent to which kcal information provision adhered to labelling recommendations. We also examined the proportion of chains that did not have point of choice kcal labelling but were able to provide kcal information on request. The study protocol was pre-registered on the Open Science Framework.

**Results:**

Of the 104 eligible chains, only a small minority (18 chains, 17%) provided in store kcal labelling. Of those that did, provision of kcal information tended not to adhere to recommended labelling practices. Of the 16 eligible chains that had previously committed to a voluntary public health pledge to provide point of choice kcal labelling, labelling did not meet recommendations and 4 (25%) did not provide kcal labelling. Of the 86 chains that did not provide kcal labelling in store, kcal information was available on request from 43 (50%) chains.

**Conclusions:**

It is rare for eating out and takeaway chains in the UK to provide point of choice kcal labelling and when labelling is provided it does not adhere to recommended labelling practices. Chains that previously volunteered to provide kcal labelling as part of an industry and public health partnership do so inadequately. Voluntary policies have not resulted in adequate kcal labelling in the UK eating out of home sector.

**Electronic supplementary material:**

The online version of this article (10.1186/s12889-019-7017-5) contains supplementary material, which is available to authorized users.

## Background

The global disease burden of obesity is substantial [1] and increases in energy intake at the population level have likely played a key role in explaining the obesity crisis [2, 3]. Consuming food served out of the home is now common. In the UK, between 25 and 39% of adults eat out in full-service or fast-food restaurants at least once a week and one in five eat takeaway meals at home on a weekly basis [4, 5]. A number of studies indicate that more frequent consumption of restaurant or takeaway meals places a person at increased risk of increased body weight [6, 7], most likely due to the lower nutritional quality and high energy density of food served outside of the home [8]. A further problem with restaurant and takeaway food is that customers tend to underestimate the number of kilocalories (kcals) in large and calorie dense meals [9, 10]. This is of particular relevance because recent research has shown that food served outside of the home is often energy dense [11]. Because of these considerations, interventions designed to improve public health may benefit from targeting the out of home food sector.

One example of a public health intervention in the out of home food sector is kcal labelling of food and drink products. Because many consumers will underestimate the number of kcals in food and drink [9, 10], legislation has now been passed in the US to ensure that kcal labelling of all food and drink products sold out of the home is provided by major catering companies [12]. The best available evidence indicates that this may have a small public health benefit because provision of kcal labelling has been shown to reduce the number of kcals ordered in restaurants [13], although this effect has not been consistently shown across all studies to date [14]. In addition, a smaller number of studies suggest that when businesses are required to provide kcal information this results in a small reduction to the kcal content of products being sold via product reformulation or replacement [15]. Therefore, although unlikely to bring about substantial improvements in population level nutrition and obesity if implemented in isolation, in combination with other policies kcal labelling has potential to improve public health.

Previous UK governments have encouraged catering chains to provide in store kcal information and in 2011, an industry and public health partnership (the Public Health Responsibility Deal) was launched that invited food industry companies to make voluntary pledges to improve public health [16]. One pledge that food industry companies could sign up to was provision of kcal labelling at point of choice in retail outlets [17]. Although the effectiveness of the Public Health Responsibility Deal has been examined more generally [18], the result of the kcal labelling pledge scheme has not been examined in detail. Moreover, there has been no examination of kcal labelling practises in the UK eating out sector. These considerations are timely because the introduction of legislation to make kcal labelling mandatory among catering chains in the UK is currently being considered by government [19]. As was the case in the US, the UK food industry may challenge any such legislation [20], on the grounds of whether action is required, whether a mandatory policy is needed and/or the feasibility of implementing kcal labelling policies.

The main aims of the present study were to examine the proportion of major UK restaurant (fast-food and full-service) and take-away chains that provide kcal information to customers in store at point of choice and the extent to which current practises adhere to kcal labelling recommendations regarding the prominence, positioning and clarity of labelling. To develop a better understanding of kcal labelling practices in the eating out sector more generally, we also sampled chain coffee shops/cafes and in store cafes of major supermarkets. In addition, we examined the adequateness of kcal labelling practises among eligible chains in the present study that also signed the Public Health Responsibility Deal (2011) kcal labelling pledge. Finally, we also examined how common it was for chains to choose not to provide in store kcal labelling but have this information available (on their websites or on request), as we reasoned this would be indicative of how easily in store labelling could be adopted.

## Methods

Our study method and analysis approach were pre-registered at https://osf.io/xy6q2/

### Chains

Based on US legislation that requires catering chains with twenty or more outlets to provide kcal labelling [21], we identified UK restaurant (including full-service and fast-food) and take-away food chains with ≥20 outlets in the UK through the use of market reports [22, 23] and accessing chain websites during August 2018. Eligible chains were coded by authors as fast-food and takeaway (as opposed to full-service) based on the following definition: chains that primarily provide consumers with largely pre-prepared ‘quick’ meals with little or no table service and/or in which take-away orders are likely to account for a significant proportion of orders [11]. Although our focus was on restaurant and take-away chains, as a kcal labelling policy would be most effective if implemented across all types of eating out establishments, we also identified and included the twenty largest coffee shop and café chains in the UK. We defined coffee shop and café chains as being chains that do not tend to provide table service and predominantly sell drinks and snacks (as opposed to full meals). In addition to this we also sampled cafes in the ‘big four’ supermarket chains in the UK (Tesco, Sainsburys, Asda, Morrisons) given that they dominate the supermarket sector in the UK, approximately 70% of market share [24], and have in store cafes in some of their outlets.

### Provision of kcal information

We used a similar methodology as in [21]. To examine whether or not a chain provided kcal information to customers at point of choice in store and/or whether this information was available on a company website or by request, we contacted each eligible chain via email (if an email address was provided) or by phone. If companies did not respond to emails we also attempted telephoning. We attempted to corroborate the chain’s response (or confirm if a chain did not provide any information) by accessing and searching the company websites of all eligible chains. In instances in which it was unclear from the chain’s response or the website whether or not kcal information was provided in store, we visited an outlet of the chain to record this. If on the basis of the chain’s response or website content we believed the chain may provide kcal information in store, we visited an outlet to confirm and examine adequateness of in store labelling. Because only a small number of supermarket chains were sampled (*n* = 4) we examined provision of kcal information by visiting a supermarket outlet.

### Quality of kcal labelling

For all chains that were providing in store kcal labelling two researchers visited an outlet of the chain (or accessed a copy of the restaurant menu from the chain website) and rated the extent to which kcal labelling adhered to recommended guidelines for best practice. Rating criteria were in line with current US kcal labelling guidelines and developed by the UK Department of Health as part of the Responsibility Deal kcal labelling pledge [25]. The two researchers rated whether:

- Kcal information was displayed at point of choice.
- Kcal information was provided for all standard food and drink items sold.
- Kcal information was provided per portion/item/meal for all meals and for all multi-portion/sharing items the number of portions is provided.
- Kcal information was positioned close to the price of the item, item description or image of the item.
- Kcal information font size and/or format was at least as prominent as the name or price.
- Reference information on kcal requirements (e.g. ‘on average women need 2,000 kcals per day’) was displayed.
- Reference information on kcal requirements was displayed clearly and prominently, so that it could be easily seen by customers.

Point of choice was operationalised as the point in the store where the main menu was located and consumers made their selections from (e.g. menu board and shelf labelling in coffee shops and cafes, handheld menu in full-service restaurants, ordering points in fast-food chains). For full coding instructions used by the two researchers, see online Additional file 1.

### Public health responsibility Deal

We identified eligible chains in the present study that had previously signed up to the Public Health Responsibility Deal kcal labelling pledge by accessing the Department of Health’s the Public Health Responsibility Deal Food Network webpage [17].

### Additional in store visits

Direct contact with chains and/or accessing chain websites allowed us to conclude, without a store visit, that a relatively large number of chains did not provide in store kcal labelling. Therefore, in addition to our pre-registered methods, to confirm the validity of this approach we made store visits to a subset of these chains (31 outlets, 45%) to confirm that kcal labelling was not provided.

## Results

We identified and included a total of 80 restaurants and take-away chains with ≥20 outlets, 58 of which were full-service restaurant chains (e.g. Pizza Express) and 22 were take-away or fast-food restaurant chains (e.g. Mcdonalds). In addition to the 20 coffee shop/cafes (e.g. Starbucks) and 4 large supermarket chains sampled, this resulted in 104 chains included in our final sample. For a full list of the individual chains included and their classification, kcal labelling provision and quality of labelling by chain, see online Additional file 1.

### Kcal labelling

We visited all four of the supermarket chains and kcal labelling was being used in three. For the remaining types of chain (full-service restaurant, fast-food and take-away, coffee shops/cafes)*,* based on contacting chains directly and/or accessing chain websites we identified: 69 did not have in store kcal labelling, 22 chains that may have in store kcal labelling, and for 9 chains we were unable to determine with confidence whether or not kcal labelling was being used. We visited outlets of the 31 chains that appeared to have in store labelling or we were unable to determine for and 15 of these chains had in store kcal labelling. Of the chains (*n* = 69) we identified as not having in store kcal labelling on the basis of direct contact with the chain and/or accessing the chain website, we visited 31 (45%) and as expected, none were using in store kcal labelling. In summary, of the 104 chains sampled, 18 chains (17%, including 3 of the supermarkets) were using in store kcal labelling. See Table 1 for kcal labelling provision by type of chain.

**Table 1:** Provision of kcal labelling in eating out chains and availability of kcal information for chains not providing in store kcal labelling

Type of chain	Number of chains	Proportion of chains providing kcal labelling^a^	Kcal information available in absence of labelling^b^
Full-service restaurant	58	3.4% (2 chains)	50% (28/56 chains)
Fast-food and takeaway	22	27.3% (6 chains)	56.3% (9/16 chains)
Coffee shop and cafes	20	35.0% (7 chains)	38.5% (5/13 chains)
Supermarket eatery	4	75.0% (3 chains)	100% (1/1 chains)
All chains	104	17.3% (18 chains)	50.0% (43/86 chains)

^a^Refers to the proportion of chains providing kcal labelling in store. ^b^ Refers to the proportion of chains that do not provide kcal labelling in store but have product kcal information available online or by request

Kcal labelling information for each individual chain is available in the online Additional file 1

### Quality of kcal labelling provision

The two researchers rating quality of kcal labelling assessed 18 chains on 7 criteria, resulting in a total of 126 ratings per researcher. Inter-rater reliability was high (95.2%) and discrepancies were resolved by a third researcher. See Table 2 for quality ratings for kcal labelling. The majority of chains provided kcal labelling at point of choice and when they did provide labelling it was close to the product name or price on the menu, as well as being provided per portion. However, the majority of chains did not provide kcal labelling for all food and drink items sold and kcal information was not presented in a prominent way. The majority of chains did not provide kcal reference information and the one chain that did provide this information did not present it in a prominent way. None of the 18 chains providing kcal labelling met all of the seven quality criteria.

**Table 2:** Quality of kcal labelling among chains providing in store kcal labelling

Type of chains providing labelling	Provided at point of choice	Provided for all items sold	Provided per portion	Provided close to product on menu	Provided prominently	Reference kcal info provided	Reference kcal info prominent
All chains providing labelling (*N* = 18)	94.4% (17 chains)	16.7% (3 chains)	100% (18 chains)	100% (18 chains)	22.2% (4 chains)	5.6% (1 chain)	0% (0 chains)
Full-service restaurant chains (*N* = 2)	100% (2 chains)	0% (0 chains)	100% (2 chains)	100% (2 chains)	100% (2 chains)	0% (0 chains)	0% (0 chains)
Fast-food and take- away chains (N = 6)	100% (6 chains)	33.3% (2 chains)	100% (6 chains)	100% (6 chains)	33.3% (2 chains)	0% (0 chains)	0% (0 chains)
Coffee shop and cafes chains (*N* = 7)	85.7% (6 chains)	14.3% (1 chain)	100% (7 chains)	100% (7 chains)	0% (0 chains)	14.3% (1 chain)	0% (0 chains)
Supermarket eatery chains (*N* = 3)	100% (3 chains)	0% (0 chains)	100% (3 chains)	100% (3 chains)	0% (0 chains)	0% (0 chains)	0% (0 chains)
Public health pledge chains (*n* = 12)^a^	100% (12 chains)	8.3% (1 chain)	100% (12 chains)	100% (12 chains)	8.3% (1 chain)	0% (0 chains)	0% (0 chains)
Non-pledge chains (n = 6)	83.3% (5 chains)	33.3% (2 chains)	100% (6 chains)	100% (6 chains)	50% (3 chains)	16.7% (1 chain)	0% (0 chains)

^a^4/16 eligible chains that signed up to the Public Health Responsibility Deal kcal labelling did not have in store kcal labelling

Kcal labelling quality ratings for each individual chain is available in the online Additional file 1

### Public health responsibility Deal chains

We identified 16 chains in the present study that signed the kcal labelling pledge. Of these 16, four did not provide in store kcal labelling. The quality of labelling provided among the remaining 12 pledge chains was similar to the full sample and none met all seven labelling quality criteria. See Table 2.

### Availability of kcal information

We identified that 43/86 (50%) of the chains that did not provide in store kcal labelling had product kcal information on their websites or were able to provide this information on request.

## Discussion

We examined in store kcal labelling practises among restaurant and take-away chains with twenty or more outlets in the UK, as well as among major coffee shop/cafes and supermarket cafes. Overall, only a minority of the 104 chains sampled provided in store kcal labelling (18 chains, 17%). Of chains that did provide in store kcal labelling, quality of labelling was not consistently in line with public health recommendations. Although chains tended to provide kcal labelling at point of choice and close to product name or price on the menu, the majority of chains did not provide kcal labelling for all items sold and kcal information tended not to be presented in a prominent way. The majority of chains also did not provide contextual information that would allow customers to interpret and use kcal labelling easily (e.g. recommended daily amount of kcals).

Provision of kcal information at point of choice when eating out of the home allows consumers to make informed dietary decisions and there is some evidence that consumers use this information to make healthier choices [13, 14]. It is therefore problematic that so few major eating out chains in the UK provide kcal labelling at point of choice. It is also likely that for kcal information to motivate healthier choices consumers need to understand the kcal information presented. For example, one US trial found that participants chose fewer kcals when kcal labels were paired with contextual information (e.g. proportion of recommended daily amount) that allowed participants to better understand the relative amount of kcals they were consuming, as opposed to no information [26]. However, the present study indicates the UK kcal labelling that is being provided tends not to be displayed prominently and without contextual information (e.g. a statement on recommended daily kcal intake), which will likely minimise usage by consumers [27]. We reasoned that chains which had previously committed to a voluntary public health pledge to provide kcal labelling may be providing kcal labelling in line with public health recommendations, but our analyses of these chains did not indicate this and we found that 4/16 (25%) chains were not using kcal labelling in the outlets of the chain that we visited. Collectively these findings suggest that voluntary policies in the UK to encourage the eating out sector to provide appropriate kcal information to customers at point of choice have not been successful [16] and as is currently being considered by UK government, a mandated policy will now be necessary.

We also examined how common it was for chains not to provide point of choice kcal labelling in store but to have this information available (via their website or on request). We were able to identify that this was the case for 50% of these chains, although this number may be larger as not all chains responded to our requests. It therefore appears that kcal labelling could be relatively easily implemented by a large proportion of major UK chains currently choosing not to provide this information at point of choice. Data from the US indicates that the overwhelming majority of eating out chains that are eligible for mandated kcal labelling have been able to implement in store labelling [21]. We did not sample smaller chains (e.g. less than 20 outlets) or independent outlets and we therefore do not know how common kcal labelling or collection of nutritional product information is among these types of businesses, but we would presume both would be less common among smaller businesses.

Strengths of the present research were that we pre-registered our study methods and analysis procedures and were able to sample a large number of eating out chains in the UK.

As noted, because of feasibility we did not examine smaller chains or independent restaurants. Because both chain and non-chain restaurants in the US have been shown to sell high kcal meals [28] we reason that kcal labelling among all catering businesses in the UK would be preferable. We used a similar methodology as in [21], using a combination of in store visits, chain website research and direct contact with chains to collect data. In addition to this it would have been ideal to visit outlets of every chain to confirm accuracy of data collection. However, we checked accuracy of instances in which we concluded a chain did not provide kcal labelling via direct contact with the chain and/or the chain’s website by visiting a subset of these chains (45%) and there were no discrepancies. Finally, although two independent researchers rated the quality of in store kcal labelling for each chain and inter-rater reliability was high, it would have been ideal to make these ratings for multiple outlets of each chain. However, menu information tends to be standardised across chain outlets, so we reason that it is unlikely that our results would differ substantially if we had adopted this approach.

### Public health relevance

The findings of the present study suggest that in the UK voluntary ‘opt-in’ policies for kcal labelling of food and drink sold in the out of home food sector result in inadequate kcal labelling practises and a mandatory policy will therefore be required for widespread and adequate adoption of kcal labelling in the out of home food sector. It will however be important to assess how effective mandatory policies are in future research, because among businesses that were providing kcal labelling in the present study labelling was often provided in a way that would be unlikely to change consumer behaviour. Although eating out of the home is becoming more common and there is public support for kcal labelling [29], given that kcal labelling will most likely have modest effects on consumer and business behaviour (e.g. reformulation), kcal labelling will need to be combined with a range of other policies to improve population level diet and nutrition.

## Conclusions

It is rare for eating out and takeaway chains in the UK to provide in store kcal labelling and when labelling is provided it does not meet recommended labelling practices. Chains that previously volunteered to provide kcal labelling as part of an industry and public health partnership do so inadequately. Voluntary policies have not resulted in adequate kcal labelling in the UK eating out of home sector.

## Additional file

Additional file 1: Supplementary materials and tables. (DOCX 30 kb)

## Data Availability

The final data sets used for analyses are available online at https://osf.io/xy6q2/

## Abbreviation

Kcal: kilocalorie
