# Supplementary material for: Point of choice kilocalorie labelling in the UK eating out of home sector: a descriptive study of major chains
Source: BMC Public Health. 2019 May 28;19:649. doi: 10.1186/s12889-019-7017-5 (PMC6540449; doi:10.1186/s12889-019-7017-5)
Supplement: Supplementary file 1 — Supplementary materials and tables. (DOCX 30 kb) [file 12889_2019_7017_MOESM1_ESM.docx]

Supplementary Materials

Quality of kcal labelling rater instructions.

1. Calorie information is displayed at point of choice. See note 1 below for more detail.
2. Calorie information is provided for all standard food and drink items sold. See note 2 below for more detail.
3. Calorie information is provided per portion/item/meal for all meals and for all multi-portion/sharing items the number of portions is provided.
4. Calorie information is positioned close to the price of the item, item description or image of the item
5. Calorie information font (size) and/or format is at least as prominent as the name or price. See note 3 below for more detail.
6. Reference information on calorie requirement (e.g. on average women need around 2,000 kcals per day) is displayed, although it does not need to accompany calorie information everywhere. See note 4 below for more detail.
7. Reference information on calorie requirement is displayed clearly and prominently, so that it can be easily seen by customers (e.g. font size and format is at least as prominent as the name or price of menu items).

Note 1. Point of choice relates to where prices are displayed and customers make their meal choices. Typical point of choice locations include (some outlets may contain multiple or all types):

Counter service outlets

• menu boards,

• counter menus;

• tickets or display information for items sold on a counter (e.g. pastries sold in a hot cabinet, or items sold in baskets on the counter),

Seated service outlets

• menus,

• table centres,

• chalk boards,

• menu boards,

Self-selection outlets

• on packaging,

• shelf edging,

• menu/tariff boards

• labelling tags (i.e. held in crocodile clips, basket sides, hanging tags etc)

Places where calorie information is given away from prices and/or that require customers to make an additional effort to obtain this information are not considered to be at point of choice.

Note 2. Standardised items means a reproducible product that is offered for at least 30 days in a year. Under certain circumstances, and for some food and drink, it may not be possible to display calorie information for each and every product for example:

• where there is too much information to display (e.g. a coffee board with multiple sizes, drinks and milks).

• if the food/drink can be customised (e.g. a sandwich deli bar).

Where potentially there is a lot of calorie information to display calorie information can be streamlined to help customers by providing calorie values for the “default option”.

Note 3. The principles state that calorie information is to be “clear and prominent” and it is for businesses to decide how to achieve this. They do not state a font, size or colour that businesses should use. This is very subjective, therefore we will base our evaluation on either the size of font used for the calorie information being the same as the name or price of the menu item or if the size of font is smaller but the format used for calorie information (e.g. font, colour) makes the information clear and prominent (i.e. it makes the information stand out). Finally, on printed menus subtler presentation than the price/menu description can be used and still be prominent because customers usually have more time to look at these menus. The information still needs to be clear and noticeable to customers.

Note 4. For example in counter service outlets, this could be done by providing one statement on the main menu board. For hand held menus this information would appear somewhere on the menu.

Table S1. Kcal labelling provision, kcal information and RD (Responsibility Deal) pledge information

| **Chain** | **Chain type** | **In store**  **Kcal labelling?** | **Kcal information available** | **RD pledge chain?** |
| --- | --- | --- | --- | --- |
| All Bar One | Full-service restaurant | N | Y | N |
| Asda | Supermarket | Y | Y | Y |
| Ask Italian | Full-service restaurant | N | Y | N |
| Bakers + Baristas | Coffee shop/café | N | N | N |
| Beefeater | Full-service restaurant | N | Y | N |
| Bella Italia | Full-service restaurant | N | N | N |
| Bill's | Full-service restaurant | N | Y | N |
| Bistrot Pierre | Full-service restaurant | N | N | N |
| Brasserie Blanc | Full-service restaurant | N | N | N |
| Brewers Fayre | Full-service restaurant | N | Y | N |
| Browns | Full-service restaurant | N | N | N |
| Burger King Holdings Inc. | Fast-food/take-away | Y | Y | Y |
| Byron | Full-service restaurant | N | N | N |
| Café Rouge | Full-service restaurant | N | N | N |
| Caffè Nero | Coffee shop/café | Y | Y | N |
| Carluccio's | Full-service restaurant | N | N | N |
| Chef & Brewer | Full-service restaurant | N | Y | N |
| Chicken Cottage | Fast-food/take-away | N | N | N |
| Chiquito's | Full-service restaurant | N | N | N |
| Chopstix | Fast-food/take-away | N | N | N |
| Coffee Republic | Coffee shop/café | N | Y | N |
| Coffee#1 | Coffee shop/café | N | Y | N |
| Costa | Coffee shop/café | Y | Y | N |
| Côte | Full-service restaurant | N | N | N |
| Dixy Chicken | Fast-food/take-away | N | N | N |
| Domino's Pizza Inc. | Fast-food/take-away | N | Y | Y |
| EAT | Coffee shop/café | Y | Y | Y |
| Eating Inn | Full-service restaurant | N | Y | N |
| Ed's Easy Diner | Full-service restaurant | N | N | N |
| Ember Inns | Full-service restaurant | N | Y | N |
| Esquires Coffee Houses | Coffee shop/café | N | N | N |
| Favourite Fried Chicken | Fast-food/take-away | N | Y | N |
| Five Guys | Fast-food/take-away | N | Y | N |
| Flaming Grill | Full-service restaurant | N | Y | N |
| Franco Manca | Full-service restaurant | N | N | N |
| Frankie & Benny's | Full-service restaurant | N | N | N |
| Gail's | Coffee shop/café | N | Y | N |
| Giggling Squid | Full-service restaurant | N | N | N |
| Giraffe World Kitchen | Full-service restaurant | N | N | N |
| Gourmet Burger Kitchen | Full-service restaurant | N | Y | N |
| Greggs | Coffee shop/café | Y | Y | Y |
| Handmade Burger Company | Full-service restaurant | N | N | N |
| Harry Ramsden | Full-service restaurant | N | N | N |
| Harvester | Full-service restaurant | N | Y | Y |
| Honest Burgers | Full-service restaurant | N | N | N |
| Hungry Horse | Full-service restaurant | N | Y | N |
| Itsu | Fast-food/take-away | Y | Y | N |
| Jamie's Italian | Full-service restaurant | N | Y | N |
| Joe & The Juice | Coffee shop/café | Y | Y | N |
| KFC | Fast-food/take-away | Y | Y | Y |
| Las Iguanas | Full-service restaurant | N | N | N |
| Leon | Fast-food/take-away | N | Y | N |
| Little Chef | Full-service restaurant | N | N | N |
| Loch Fyne | Full-service restaurant | N | Y | N |
| Lounges | Full-service restaurant | N | N | N |
| Marco Pierre White Steakhouse Bar & Grill | Full-service restaurant | N | Y | N |
| McDonald’s Corp | Fast-food/take-away | Y | Y | Y |
| Miller & Carter | Full-service restaurant | N | N | N |
| Morrisons | Supermarket | N | Y | Y |
| Muffin Break | Coffee shop/café | N | N | N |
| Nando's | Full-service restaurant | N | Y | N |
| Old English Inns | Full-service restaurant | N | Y | N |
| O'Neill's | Full-service restaurant | N | N | N |
| Papa John's International Inc. | Fast-food/take-away | N | Y | N |
| Patisserie holdings (Patisserie Valerie) | Coffee shop/café | N | N | N |
| Paul depuis 1889 | Coffee shop/café | N | Y | N |
| Pepe’s Piri Piri | Fast-food/take-away | N | N | N |
| Pho café | Full-service restaurant | N | Y | N |
| Pizza Express | Full-service restaurant | N | Y | N |
| Pizza GoGo Ltd. | Fast-food/take-away | N | N | N |
| Pizza Hut | Fast-food/take-away | N | Y | Y |
| Pret a Manger | Coffee shop/café | Y | Y | Y |
| Prezzo | Full-service restaurant | N | N | N |
| Puccino's | Coffee shop/café | N | N | N |
| Sainsbury’s | Supermarket | Y | Y | Y |
| Sayers | Coffee shop/café | N | N | N |
| Sizzling Pubs | Full-service restaurant | N | Y | N |
| Slug and Lettuce | Full-service restaurant | N | N | N |
| Starbucks | Coffee shop/café | Y | Y | Y |
| Stonehouse Pizza & Carvery | Full-service restaurant | N | Y | N |
| Subway | Fast-food/take-away | Y | Y | Y |
| Table Table | Full-service restaurant | N | Y | N |
| Taco Bell | Fast-food/take-away | N | Y | N |
| Tesco | Supermarket | Y | Y | Y |
| TGI Fridays | Full-service restaurant | N | N | N |
| The Cornish Bakery | Coffee shop/café | N | N | N |
| Toby Carvery | Full-service restaurant | N | Y | N |
| Tops Pizza | Fast-food/take-away | N | N | N |
| Tortilla | Fast-food/take-away | N | Y | N |
| Turtle Bay | Full-service restaurant | N | N | N |
| Upper Crust (SSP UK) | Coffee shop/café | N | N | N |
| Vintage Inns | Full-service restaurant | N | Y | N |
| Wagamama | Full-service restaurant | N | Y | N |
| Wahaca | Full-service restaurant | N | N | N |
| Walkabout | Full-service restaurant | N | Y | N |
| Wasabi | Fast-food/take-away | N | Y | N |
| West Cornwall Pasty Co. | Coffee shop/café | N | Y | N |
| Wetherspoons | Full-service restaurant | Y | Y | N |
| Wildwood | Full-service restaurant | N | N | N |
| Wimpy | Fast-food/take-away | Y | Y | N |
| Wok&Go | Fast-food/take-away | N | N | N |
| Yates | Full-service restaurant | N | Y | N |
| YO! Sushi | Full-service restaurant | Y | Y | Y |
| Zizzi | Full-service restaurant | N | Y | N |

Table S2. Kcal labelling quality ratings for individual chains

| **Chain** | **Provided at point of choice** | **Provided for all items sold** | **Provided per portion** | **Provided close to product on menu** | **Provided prominently** | **Reference kcal info provided** | **Reference kcal info prominent** |
| --- | --- | --- | --- | --- | --- | --- | --- |
| ASDA Café | Y | N | Y | Y | N | N | N |
| Burger King | Y | N | Y | Y | N | N | N |
| Café Nero | Y | N | Y | Y | N | N | N |
| Costa | N | N | Y | Y | N | N | N |
| Eat | Y | N | Y | Y | N | N | N |
| Greggs | Y | N | Y | Y | N | N | N |
| Itsu | Y | N | Y | Y | Y | N | N |
| JD Weatherspoons | Y | N | Y | Y | Y | N | N |
| Joe and the Juice | Y | Y | Y | Y | N | Y | N |
| KFC | Y | N | Y | Y | N | N | N |
| Mcdonalds | Y | Y | Y | Y | N | N | N |
| Pret | Y | N | Y | Y | N | N | N |
| SAINSBURYS cafe | Y | N | Y | Y | N | N | N |
| Starbucks | Y | N | Y | Y | N | N | N |
| Subway | Y | N | Y | Y | N | N | N |
| TESCO | Y | N | Y | Y | N | N | N |
| WIMPY | Y | Y | Y | Y | Y | N | N |
| Yo Sushi | Y | N | Y | Y | Y | N | N |
